# Associations of self-repression with disordered eating and symptoms of other psychopathologies for men and women

**DOI:** 10.1186/s40337-022-00569-y

**Published:** 2022-03-21

**Authors:** Rachel Bachner-Melman, Yonatan Watermann, Lilac Lev-Ari, Ada H. Zohar

**Affiliations:** 1 Clinical Psychology Graduate Program, Ruppin Academic Center, Emek Hefer, Israel; 2 School of Social Work, Hebrew University of Jerusalem, Jerusalem, Israel; 3 The Lior Tsfaty Center for Suicide and Mental Pain Studies, Ruppin Academic Center, Emek Hefer, Israel

**Keywords:** Self-repression, Disordered eating, Selflessness, Concern for appropriateness, Cross-situational variability, Attention to social comparison information, Gender differences

## Abstract

**Background:**

Disordered eating has been found to be associated with constructs involving self-repression, such as selflessness (the tendency to relinquish one’s needs for others’), and concern for appropriateness (an alertness to information about social comparison and tendency to vary one’s behavior in different social situations). This study aimed to examine associations between these self-repression variables and symptoms of general psychopathology for women and men in a community sample.

**Methods:**

Two hundred and thirty-six participants (92 men) aged 18–76 (M = 29.11 ± 10.10) volunteered to complete online measures of disordered eating, concern for appropriateness (cross-situational variability and attention to social comparison information), selflessness, and symptoms of depression, anxiety and somatization. Structural equation models were built to assess pathways between the study variables for men and women separately.

**Results:**

A MANOVA 2*7 design showed that women scored significantly higher than men on measures of selflessness, disordered eating and depression. For men, selflessness scores were positively and significantly associated only with depression scores. Cross-situational variability scores were positively associated with depression, somatization and anxiety scores. For women, selflessness scores were positively and significantly associated with depression, disordered eating, somatization and anxiety scores. Cross-situational variability scores were positively and significantly associated with depression, anxiety and somatization scores but not with disordered eating scores. Attention to Social Comparison Information scores were positively and significantly associated only with disordered eating scores.

**Conclusions:**

Self-repression is more closely linked to psychopathology in women than in men. For men, self-suppression seems to be associated with symptoms of internalizing disorders, but not disordered eating. Even for women, it appears that self-repression is not connected exclusively with disordered eating, but with symptoms of psychopathology in general. Future research should explore why self-suppression plays such a central role in women’s psychopathology.

## Plain English summary

Disordered eating has been found to be associated with self-repression, specifically with selflessness, the tendency to relinquish one’s needs for others’, and concern for appropriateness, an alertness to information about social comparison and tendency to vary one’s behavior in different social situations. This study aimed to examine links between these self-repression concepts and symptoms of eating disorders, depression, anxiety and somatization for women and men. Two hundred and thirty-six participants completed online questionnaires and models were built to assess pathways between the study variables for men and women separately. Women reported more selflessness, disordered eating and depressive symptoms than men and the pattern of connections between all variables was very different for men and for women. Self-repression is a more closely linked to psychological disturbances in women than in men. For men, self-repression is not linked to disordered eating, as it is for women. However even for women, self-repression is linked not exclusively with disordered eating, but also with symptoms of anxiety, depression and somatization. Future research should explore why self-repression is so central to women’s psychological problems.

## Introduction

Disordered eating has been found to be associated with deficiencies in the sense of self [1], with cross-sectional data indicating that young men and women who struggle to define their beliefs about themselves tend to have a greater propensity to engage in disordered eating than those who do not [2]. People with eating disorders have historically been described psychologically as lacking a solid sense of self. For example, Hilda Bruch [3, p56] saw "over-submissiveness, abnormal considerateness, and lack of self-assertion" as central characteristics of her patients with anorexia that fed and sustained their disorder. A weak sense of self expressed in submission and non-assertiveness has been attributed to eating disorder patients within interpersonal relationships in general [4, 5] and within the family specifically [6, 7]. From the perspective of self-psychology, eating disorder patients experience themselves as selfless beings who serve others [8, 9]. The subjective self of people with eating disorders also involves guilt [10], suffering [11], physiological self-punishment and self-sacrifice [12].

From a cognitive and sociological perspective, Geller et al. [13] observed the tendency of women with anorexia to ‘silence their self’, to secure interpersonal relationships by inhibiting their self-expression [13]. Zaitsoff et al. [14] found anger inhibition and “silencing the self” to be associated with disordered eating in a sample of over 200 female high school students. An association was also observed in a nonclinical population between drive for thinness and “narcissistically abused personality”, defined as placing others’ needs before one’s own [15]. Similar concepts that have been examined in relation to disordered eating include “pathological altruism” [16, 17] and “pathological concern” [18]. Bardone-Cone et al. [19] provided a comprehensive overview of self-related constructs and deficits in relation to eating disorders and disordered eating.

Deficits in the self have also been found to be connected to other forms of psychopathology [20, 21]. For example, depression has been found to be associated with an inner sense of emptiness and deadness [22], and a weak sense of self has been linked to symptoms of anxiety disorders [23, 24] and to somatization [25].

Two variables that have been studied empirically in the context of an association between disordered eating and self-repression are selflessness [26, 27] and concern for appropriateness, or protective self-presentation [28]. Selflessness is the tendency to relinquish one’s own interests and ignore one’s genuine needs in the service of others’ interests and well-being. Although this characteristic was conceptualized, defined and quantified in the context of patients with clinical eating disorders [26], an association between measures of disordered eating in the general population and scores on the Selflessness Scale (SS) [26] has consistently been found in research [26, 27, 29–31]. Selflessness has even been shown to predict the development of disordered eating over time [30]. The price tag of selflessness is high, since it leads people to relinquish their own interests, forfeit their wellbeing and deny the fulfillment of their interpersonal needs. The toll of selflessness and its link with disordered eating may be due in part to an association between eating pathology and the frustration of psychological needs [32], whereby people whose psychological needs are not met focus on meeting those of others, as a form of self-sacrifice.

Concern for appropriateness, measured by the Concern for Appropriateness Scale [33] is a protective self-presentation style adopted to avoid social disapproval and a sense of failure in interpersonal relations [33]. The two concepts measured by its subscales are cross-situational variability and attention to social comparison information. Unlike selflessness, which was formulated in the context of eating pathology, concern for appropriateness hails from the domain of social psychology. People high on this trait tend to experience high levels of negative emotions such as shame and embarrassment [34] and to comply with external guidelines and authorities [35]. Scores on the Concern for Appropriateness Scale have been found to correlate negatively with individuation, self-esteem, extraversion, and emotional stability [36], and positively with perfectionism [36] and maladaptive social behavior [33]. It follows from these findings from the social psychological literature that link concern for appropriateness with negative emotional states, that concern for appropriateness should be of interest in the field of mental health and psychopathology.

Research using concern for appropriateness in the field of consumer and marketing has shown an association with the degree to which individuals are influenced by other individuals, society and the mass media. People high on concern for appropriateness tend to be high on consumer conformity [37] and susceptibility to peer pressure [38]. They were found to be strongly influenced by messages promoting the avoidance of social risk [39, 40]), to be particularly concerned with others’ reactions, and to be very sensitive to social and cultural influences including mass communication [41, 42].

Since eating disorder symptoms have been linked to high interpersonal sensitivity, inappropriately submissive behavior and accommodation to others [43, 44], the findings from social psychology and consumer and marketing psychology mentioned feed into both to the concept of selflessness and to the internalization of the thin body ideal promulgated by the media. Selflessness was defined according to the theory of self-psychology [26] as the tendency of people with eating disorders to ignore one’s own needs and fulfill those of others. This tendency necessitates a constant and alert monitoring of one’s social circumstances, which links right in with the concept of concern for appropriateness. The unquestioning acceptance and internalization of women’s need to be thin, marketed aggressively by the media throughout the Western world, leads many girls and women along the path of body dissatisfaction, dieting and food restriction in an attempt to make their body conform to the marketed ideal [45]. A significant association between scores on the Concern for Appropriateness Scale and measures of disordered eating have indeed been consistently found in studies on eating pathology [28, 46, 47]. These studies suggest that people with high concern for appropriateness may strongly internalize social norms for appearance and thinness and strive to adapt their bodies to fit them via disordered eating.

Concern for appropriateness has also been examined in relation to several measures of general psychopathology. For example, positive and significant associations were found between concern for appropriateness and maladaptive social behavior [33], perfectionism, negative emotions, low self-esteem and emotional instability [36]. It is therefore possible that this characteristic also underlies symptoms of psychopathology other than eating pathology. Yet associations between selflessness and symptoms of psychopathology other than eating disorders have hardly been explored to date.

The specific subscales of the Concern for Appropriateness Scale may be of interest to examine in regard to symptoms of eating disorders and other psychopathologies. Cross-Situational Variability (CSV) is an active tendency to adapt one’s behavior to the perceived expectations of one’s social environment [33], whereas Attention to Social Comparison Information (ASCI) is a more passive tendency to be on the constant lookout for signs in one’s social environment about behavioral guidelines and expectations. Despite a strong connection between the ASCI subscale and sensitivity to social and cultural influences [42], ASCI was not found to contribute to the intention to exercise and eat healthily [48].

Another factor relevant to the connection between disordered eating and both measures of self-deficiency that remains to be explored is gender. Whereas a connection between selflessness and disordered eating has been consistently observed in women [26, 27, 29–31], no published studies to date have examined selflessness and disordered eating in men. Nor has gender been adequately examined in relation to the connection between disordered eating and concern for appropriateness. Whereas most published research presenting this association has focused on women [28], two studies found significant correlations between concern for appropriateness and disordered eating in males [46, 47, unpublished data]. The association of these self-repression constructs with symptoms of psychopathology other than eating disorders remains to be clarified.

This study aims to extend our understanding of these associations beyond the consistent finding that self-repression variables relate to eating disorders in women. We examine whether there is a significant association between self-repression and disordered eating in a community sample of men and women in Israel, over and above the presence of symptoms of other psychopathologies. This is important because self-psychology postulates that self-repression is associated specifically and uniquely with disordered eating and eating disorders [28, 30]. So is self-repression related profoundly and exclusively with disordered eating, or is a poorly articulated self rather a risk factor for psychopathology in general? Examining the boundaries of the prediction from self-psychology theory may lead to a deeper understanding of psycho-pathogenesis for both genders. Since research on disordered eating in men has lagged behind research in women, we will examine the association of the subscales of Concern for Appropriateness Scale and selflessness with eating disorder symptoms and other pathology clusters in a community sample of both men and women. Since cultural pressures to be thin tend to target girls and women specifically, we expected to find stronger associations between self-repression and symptoms of eating disorders and other psychopathologies in women than in men.

Our hypotheses were as follows:

1. The associations between selflessness, cross-situational variability, attention to social comparison information, disordered eating, depression, anxiety and somatization would differ significantly between men and women, with a tendency towards stronger associations for women.
2. Comprehensive models assessing the connections between age, selflessness and concern for appropriateness subscales with psychopathology (depression, anxiety, somatization and disordered eating) would differ significantly for men and women.

## Methods

### Participants

Two hundred and thirty-six participants (144 women, 92 men) aged 18–76 (M = 29.11, SD = 10.10) participated in the study. Of these, 128 (78 men) were recruited via social media and 108 (14 men) were undergraduate students who received course credit in exchange for participation. Two $50 gift cards were raffled among the participants recruited via the social media. To compare the demographic characteristics of men and women, chi square tests of independence and T-tests for independent samples were run (see Table 1). There were no significant gender differences for mother tongue, religion or sexual orientation. However, the men were significantly older and heavier than the women. They also had more years of education and were more frequently married, no doubt as a consequence of the age difference.

**Table 1:** Participants’ demographic characteristics and gender comparison

	Men (n = 92)	Women (n = 144)	Whole sample (N = 236)	Significance
Age	M = 35.36 + 12.50	M = 25.12 + 5.20	M = 29.11 + 10.10	t = 8.73***
BMI	M = 24.48 + 3.25	M = 22.71 + 3.69	M = 23.4 + 3.62	t = 3.77***
Mother tongue	92.4% Hebrew 2.1% Arabic 2.1% Russian 3.3% other	91.7% Hebrew 2.6% Arabic 2.4% Russian 3.3% other	91.9% Hebrew 2.5% Arabic 2.3% Russian 3.3% other	χ^2^ = 0.04
Religion	94.6% Jewish 2.2% Muslim 3.2% other	97.2% Jewish 2.8% Muslim	96.2% Jewish 2.5% Muslim 1.3% other	χ^2^ = 1.08
Education	31.5% high school 33.7% BA 25% MA 9.8% other	63.9% high school 25% BA 6.9% MA 4.2% other	51.2% high school 28.4% BA 14% MA 6.4% other	χ^2^ = 34.94***
Sexual orientation	96.7% heterosexual 1.1% homosexual 1.1% bisexual 1.1% other	95.8% heterosexual 2.1% lesbian 0.7% bisexual 1.4% other	96.1% heterosexual 1.7% homosexual 0.9% bisexual 1.3% other	χ^2^ = 0.46
Marital status	39.1% single 46.7% married 10.9% in a relationship 3.3% other	71.5% single 11.8% married 16% in a relationship 0.7% other	58.9% single 25.4% married 14% in a relationship 1.6% other	χ^2^ = 41.23***

*BMI* body mass index

^***^p < .001 (2-tailed)

### Measures

Demographic characteristics included age, gender, weight, height, mother tongue, sexual orientation, education, religion, and marital status.

Selflessness was measured by the Selflessness Scale [26], a 15-item questionnaire assessing the tendency to relinquish one's own interests and ignore one's genuine needs in the service of others' interests and well-being. Responses are recorded on a four-point scale from 1 (not at all true) to 4 (very true). The original version, used in this study, was written in Hebrew [26]. Sample items are “I am willing to sacrifice a lot for the benefit of others” and “I am more bothered by other's problems than my own.” Construct validity for the original Hebrew Selflessness Scale was demonstrated via exploratory factor analysis with over 1000 participants, yielding four factors: sacrifice for family, sacrifice for others, self-denial and lack of self-interest [27]. The scale’s test–retest reliability was 0.93 [26]. The Selflessness Scale has demonstrated adequate reliability, with Cronbach's alpha falling between 0.61 and 0.66; [26, 27]. Cronbach's alpha in this study was 0.68.

Concern for appropriateness was measured by the Concern for Appropriateness Scale [33], a 20-item questionnaire assessing a protective self-presentation style characterized by a passive and withdrawn social orientation intended to avoid social disapproval and a sense of interpersonal failure. Responses are scored on a six-point scale from 1 (not at all true) to 6 (very true). This questionnaire has been validated in English [49], as well as in Hebrew using 1,010 females and 284 males [28]. The validated Hebrew version was used in this study. The Concern for Appropriateness Scale has two subscales: (1) Cross-Situational Variability, or the extent to which people adapt their behaviors to the social norms or expectations of others (e.g. “In different situations and with different people, I often act like very different persons”); and (2) Attention to Social Comparison Information, or the extent to which people monitor others’ behaviors for behavioral guidelines (e.g. “I try to pay attention to the reactions of others to my behavior in order to avoid being out of place”). Cronbach's alpha varies between 0.82 and 0.89 [49] and was 0.88 for the total score in this study (0.84 for each of the subscales).

Eating disorder symptoms were measured by the Eating Disorder Examination Questionnaire [50], a self-report version of the Eating Disorder Examination [51]. The Eating Disorder Examination Questionnaire is a 28-item questionnaire assessing the severity of eating disorders symptoms based on Diagnostic and Statistical Manual of Mental Disorders-5 (DSM-5; [52]) criteria. The first 22 items are scored on a seven-point scale between 0 (I do not agree at all) and 6 (I completely agree) and the other six require an open numerical reply indicating the frequency of weight, shape, and purging behaviors in the last 28 days. These six items are used for diagnostic purposes and are not included in scores for analyses. A validated Hebrew translation with good psychometric properties was used [53]. Cronbach's alpha for the Eating Disorder Examination Questionnaire in this study was 0.96.

Symptoms of depression were measured by the Patient Health Questionnaire-9 [54], a 9-item questionnaire based on the DSM-5 [52] assessing the frequency of symptoms of depression during the past two weeks. Responses are scored on a four-point scale between 0 (not at all) and 3 (almost every day). In this study a Hebrew translation of the Patient Health Questionnaire-9 used in previous research was employed [55]. Cronbach's alpha varies between 0.86 and 0.89 [54] and was 0.87 in this study.

Symptoms of anxiety and somatization were measured by the Brief Symptom Inventory-18 [56] anxiety and somatization subscales (six items in each subscale). The third subscale that was not used in this study assesses depression. Respondents mark to what extent they experienced specific symptoms of anxiety and somatization during the past two weeks on a five-point scale from 0 (not at all) to 4 (extremely). In this study a Hebrew translation of the Brief Symptom Inventory-18 that showed good psychometric properties in previous research [57] was used. Cronbach's alphas for these subscales in Derogatis’ [56] original scales were 0.79 for anxiety and 0.74 for somatization. In this study they were 0.87 and 0.80 respectively.

### Procedure

The study was approved by the Ruppin Academic Center Ethics Committee. Participants received an online link to the questionnaire. All participants were above 18 and provided informed consent. Participants were first asked to answer demographic questions and to provide their e-mail address if they wished to participate in a raffle for a prize. The Selflessness Scale, Concern for Appropriateness Scale, Eating Disorders Examination-Questionnaire, Patient Health Questionnaire-9 and Brief Symptom Inventory-18 anxiety and somatization subscales were then presented in randomized order.

Data was analyzed using SPSS 22 and AMOS 26. A Multivariate analysis of variance (MANOVA) was used to test for gender differences. Pearson correlations were calculated to examine associations between the variables. Fisher Z-transformations were used to compare correlations between groups. Structure Equation Modeling (SEM) was utilized to assess the different associations between selflessness, cross-situational variability and attention to social comparison information with the different pathologies in regarding men and women. A power analysis was conducted, including all variables, with predicted effect size of 0.3, alpha error probability of 0.05 and confidence level of 0.95. The recommended sample size was 239.

## Results

### Comparison of demographic variables between men and women

The mean age of the men in our sample (M = 35.36, SD = 12.50) was significantly greater than the mean age of the women (M = 25.12, SD = 5.20; t_(234)_ = 8.73, *p* < 0.001; *Cohen’s d* = 1.0). We therefore entered age as a covariate in all further analyses. Men were also significantly more educated (65.8% had tertiary education) than women (66.7% had high school education only; ${\chi }_{(2)}^{2}=27.13, p<.001; \eta =.35$) and were significantly more likely than women to be married or in a significant relationship (59.5% and 28%, respectively; ${\chi }_{(2)}^{2}=38.18, p<.001$). Higher education was associated with age (F_(2,217)_ = 32.23, *p* < 0.001; ${\eta }^{2}=.23)$ as was marital status (F_(2,229)_ = 80.41, *p* < 0.001; ${\eta }^{2}=.41)$. Since these variables were categorial rather than continuous, they were not held constant in the analyses. No significant differences between women and men were observed for religion.

**Fig. 1:**
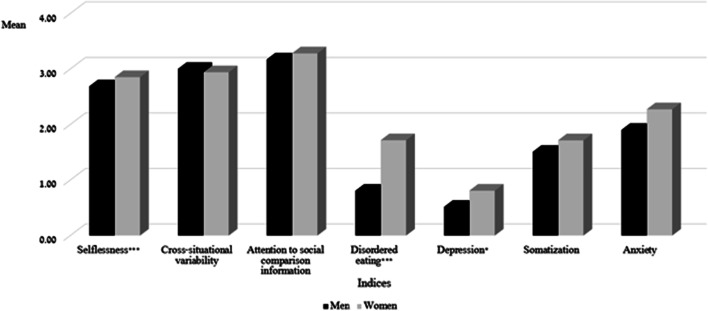
Gender differences for study variables (N = 227). *p < .05; **p < .01; ***p < .001

A MANOVA 2*7 design was then used to compare men and women on all study indices with age entered as a covariate: selflessness, cross-situational variability, attention to social comparison information, disordered eating, depression, anxiety and somatization. Gender was the independent variable and study variables were the dependent variables. Significant gender differences were observed (F_(7,218)_ = 4.84, *p* < 0.001;${\eta }^{2}=.41$; see Fig. 1). Women scored significantly higher than men on selfessness (F _(1,224)_ = 16.51, *p* < 0.001$; {\eta }^{2}=.07$), the EDE-Q (F_(1,224)_ = 17.12, *p* < 0.001;${\eta }^{2}=.07$) and depression (F_(1,224)_ = 3.84, *p* = 0.05;${\eta }^{2}=.02$). No significant differences were observed for anxiety or somatization.

#### Hypothesis 1:

The associations between selflessness, cross-situational variability, attention to social comparison information, disordered eating, depression, anxiety and somatization would differ significantly between men and women, with a tendency towards stronger associations for women.

To assess the associations between selflessness, cross-situational variability, attention to social comparison information, disordered eating, depression, anxiety and somatization scores separately for men and women, two-sided Pearson correlations were calculated, with age entered as a covariate (see Table 2). For women, selflessness scores correlated significantly and positively with attention to social comparison information (not cross-situational variability), disordered eating, depression, anxiety and somatization scores. For men, the only significant association was with the attention to social comparison information. For women, both Concern for Appropriateness subscale scores (cross-situational variability and attention to social comparison information) were positively and significantly associated with disordered eating, depression, anxiety and somatization scores. For men, attention to social comparison information scores were positively and significantly associated with selflessness scores but none of the other study variables. Cross-situational variability scores were positively and significantly associated with depression and anxiety scores.

**Table 2:** Pearson correlations between study variables (age entered as a covariate)

Women (Men)	Cross-situational variability	Attention to social comparison information	Disordered eating	Depression	Somatization	Anxiety
Selflessness	.02 (− .09)	.22** (.22*)	.35*** (.02)	.17* (.14)	.21* (-.01)	.21* (-.01)
Cross-situational variability		.46*** (.41***)	.20* (.17)	.55*** (.24*)	.41*** (.20)	.36** (.23*)
Attention to social			.32*** (.20)	.31*** (.06)	.24** (.005)	.28*** (.07)
Comparison information						
Disordered eating				.48*** (.40***)	.27** (.34***)	.34*** (.28**)
Depression					.59*** (.73***)	.66*** (.72**)
Somatization						.72*** (.73***)

BOLD = significant differences between correlations

^*^p < .05; **p < .01; ***p < .001

Fisher’s Z was calculated to compare correlations for men with correlations for women. Significant gender differences were found between the following correlations: selflessness—disordered eating (*p* < 0.001); cross-situational variability—depression (*p* < 0.001); cross-situational variability—somatization (*p* < 0.05); attention to social comparison information—depression (*p* < 0.05); attention to social information—somatization (*p* < 0.05); depression – somatization (*p* < 0.05).

#### Hypothesis 2:

Comprehensive models assessing the connections between age, selflessness and concern for appropriateness subscales with psychopathology (depression, anxiety, somatization and disordered eating) would differ significantly for men and women.

**Fig. 2:**
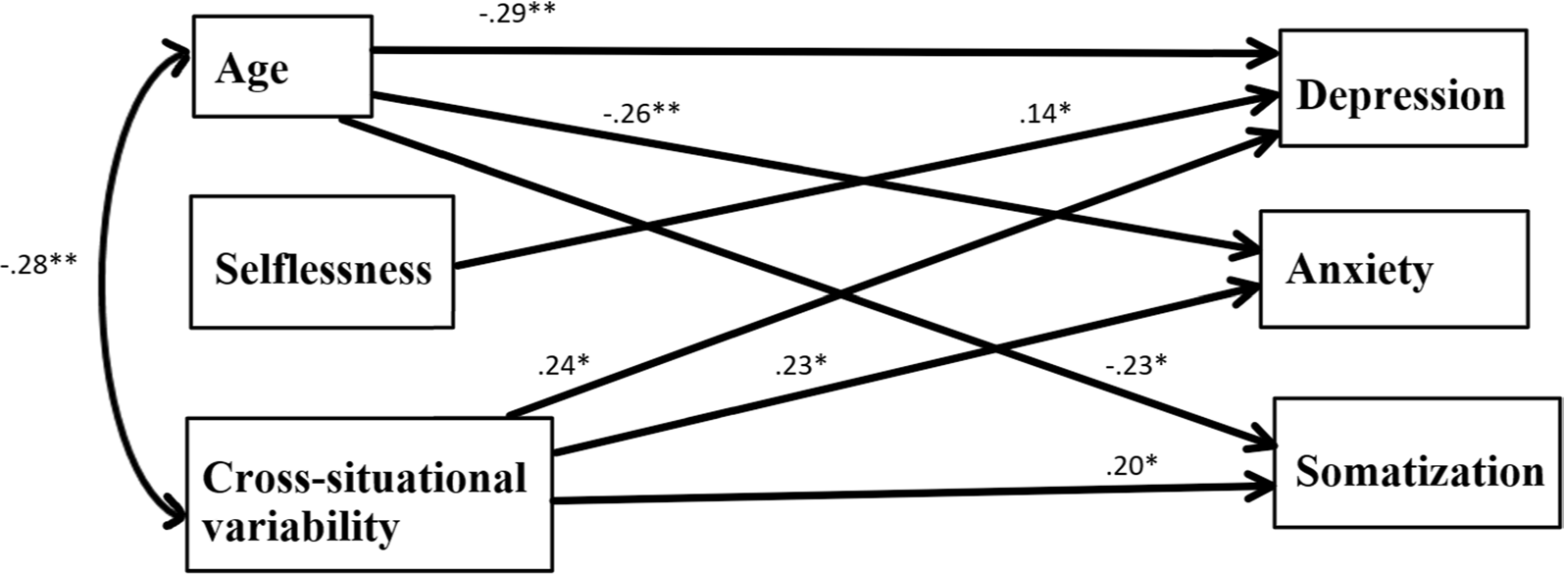
SEM model depicting the associations of selflessness and the Concern for Appropriateness Scale subscales with measures of psychological pathology for men. *p < .05; **p < .01; ***p < .001

**Fig. 3:**
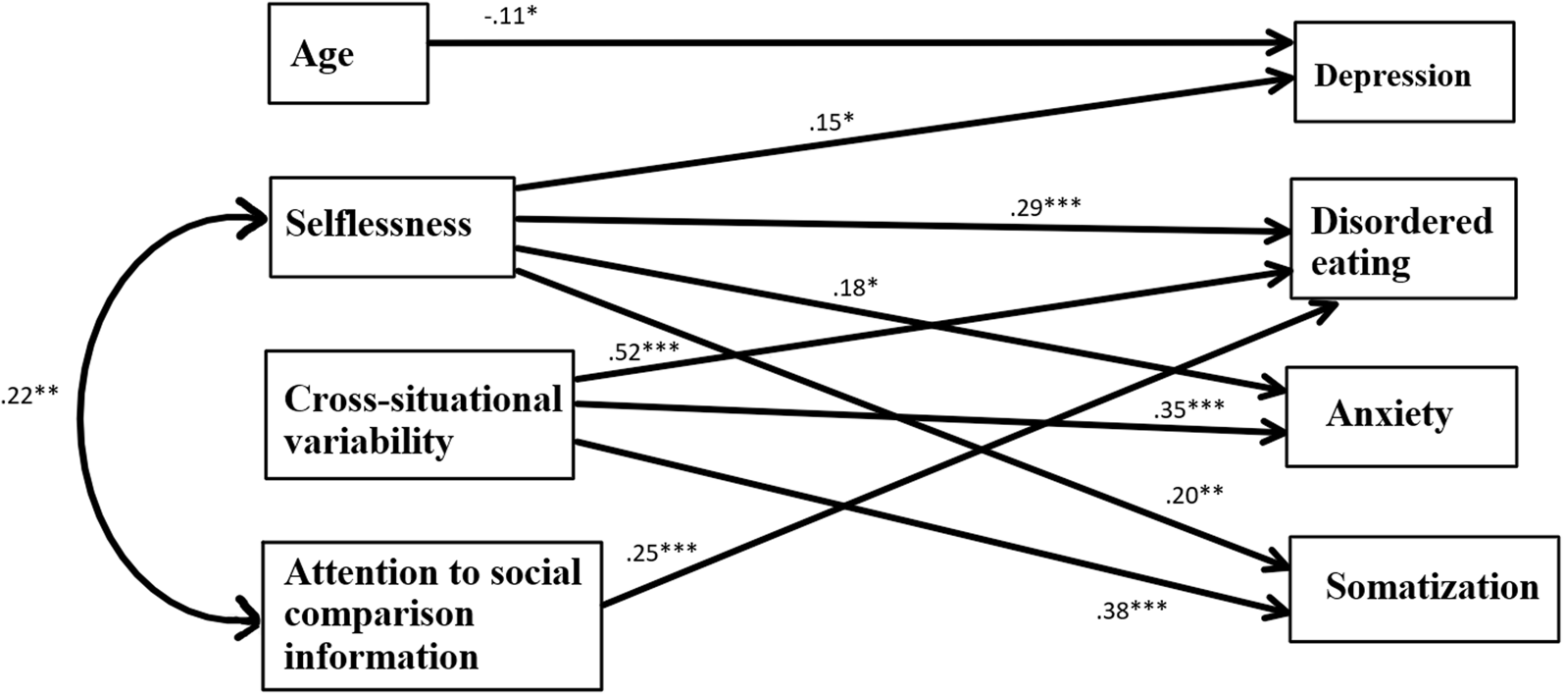
SEM model depicting the associations of selflessness and the Concern for Appropriateness Scale subscales with measures of psychological pathology measures for women. *p < .05; **p < .01; ***p < .001.

Two structural equation models (SEM) were designed to assess the multiple pathways between 1. Selflessness and Concern for Appropriateness Scale subscale scores (cross-situational variability and attention to social comparison information and 2. psychological pathology measures (disordered eating, depression, anxiety and somatization) for women and for men separately, while controlling for age. As a combined rule for the acceptance of our model, we chose the following acknowledged values: normed fit index (NFI) > 0.90 [58] and root mean square error of approximation (RMSEA) < 0.08 ([59]; see Figs. 2, 3). Figure 2 shows that for men, the Chi Square goodness-of-fit index presented an excellent fit for the data, ${\chi }_{(4)}^{2}$=2.07, *p* = 0.72); NFI = 0.99; CFI = 1.00; RMSEA = 0.00; standardized root means square residual (RMR) = 0.0**4.**

For men, none of the dependent variables were associated with disordered eating, which was therefore removed from the model. Attention to social comparison information was significantly associated with no other indices, so was also removed. Age was negatively correlated with all psychopathology indices, so that the older the men, the less psychopathology they had. Selflessness was positively and significantly associated only with depression, so that the more selfless the men, the more depressed they were. Cross-situational variability scores was positively associated with depression, somatization and anxiety. Figure 3 shows that for women, the Chi Square goodness-of-fit index presented an excellent fit for the data (${\chi }_{(12)}^{2}$=12.42, *p* = 0.41); NFI = 0.97; CFI = 0.99; RMSEA = 0.02; standardized root means square residual (RMR) = 0.0**6.**

For women, age was significantly and negatively associated only with depression, so that the older the women, the less depressed they were. Selflessness was positively and significantly associated with disordered eating, depression, somatization and anxiety, so that more selfless the women, the more symptoms of disordered eating, depression, somatization and anxiety they displayed. It is important to note that selflessness was significantly associated with disordered eating for women, but not for men. For women, cross-situational variability was positively and significantly associated with disordered eating, depression, anxiety and somatization but not with depression. Attention to social comparison information was positively and significantly associated with disordered eating only.

## Discussion

This study examined gender differences in the links between self-repression (selflessness, concern for appropriateness) and symptoms of various psychopathologies (eating disorders, depression, anxiety and somatization) in a non-clinical population. Gender differences were found for some of the variables examined. Three psychological constructs of self-repression were included. The first two were measured by Lennox and Wolfe’s [33] Concern for Appropriateness Scale: Cross-situational variability, a chameleon-like tendency to monitor and adapt actively but defensively to perceived expectations of one’s changing social environment; and attention to social comparison information, a defensive but more passive vigilance for clues in one’s social environment about behavioral guidelines and expectations. No significant sex differences were found for these variables, which is consistent with previously published findings [16, 60]. The third psychological construct of self-repression was selflessness, the tendency to ignore one’s own interests and needs for the benefit of others’ well-being. Selflessness, a form of pathological altruism theoretically related to anorexia nervosa [26], has not been studied extensively and to the best of our knowledge there are no previous reports of gender comparisons. In our study women reported significantly higher levels of selflessness than men. This is consistent with a large body of research indicating that females tend to be more altruistic [61, 62] and self-sacrificing [63] than males. In male-dominated societies, women may relinquish their own interests for others more readily than men [64].

Concerning symptoms of psychopathology, women reported significantly higher levels of depressive symptoms than men, which is consistent with a plethora of previous studies and meta-analyses [65]. Women also reported higher levels of disordered eating than men, as has been consistently reported in the literature (e.g. [66]). However, we observed no gender differences for somatization or anxiety symptoms, despite evidence of a higher prevalence of anxiety and somatization disorders in women than in men [67].

Of central interest in our findings, however, are the significant differences between men’s and women’s patterns of intercorrelations between study variables—more specifically, the different patterns of associations observed for men and women between self-repression variables and psychopathology variables. In the SEM model built using men’s data only, disordered eating was significantly associated with no other study variables and was consequently dropped from the model.

Two of the three self-repression variables, selflessness and cross-situational variability, were related to symptoms of other psychopathologies, and these associations were driven more by active, chameleon-like “fitting in” (cross-situational variability) rather than by the more passive observation of contextual social cues (attention to social comparison information). More specifically, for men selflessness and cross-situational variability were significantly associated with depressive symptoms, and cross-situational variability was associated in addition to symptoms of anxiety and somatization. Attention to social comparison information, which involves the active cognitive monitoring of contextual social cues but a passive behavioral stance, was unrelated to psychopathology symptoms of all kinds. For men, self-repression therefore seems to be associated with symptoms of internalizing disorders, but to be neither a route to nor consequence of disordered eating.

A very different picture emerged for women, for whom self-repression seems to play a much more central and powerful role in psychopathology, including disordered eating, than it does for men. In the SEM model built using women’s data only, cross-situational variability, an active, defensive adaptation to social cues, was related to symptoms of depression, anxiety and somatization but not to disordered eating. In contrast, the more passive observation of societal expectations, attention to social comparison information, was related only to disordered eating and not to symptoms of other psychopathologies. Selflessness was significantly related to all four domains of psychopathology examined in our study: symptoms of eating disorders, depression, anxiety and somatization. Selflessness and the Selflessness Scale [26] were conceived and built within the self-psychology theoretical viewpoint towards eating disorders. According to this approach, people with eating disorders sacrifice their needs and starve themselves because of a poor sense of self [26]. The significant association consistently observed between selflessness and disordered eating [16, 29, 31] therefore comes as no surprise. But why does this not appear to be true for men?

Men’s response to self-suppression seems to be channelled towards other domains of psychopathology, possibly because contemporary social influences cause their self-perception to be less closely linked to their body image [68]. Fasting as self-sacrifice has been documented as a predominantly female phenomenon, dating back as far as thirteenth century to what was viewed as a saintly condition termed anorexia mirabilis [69]. Fasting and self-sacrificial women continued to be perceived as miraculous, with starving heroines featured in German literature from the eighteenth century [70] and a shift from “sainthood” to “patienthood” occurring in the early nineteenth century with the development of a medical model of eating disorders [71].

Palazzoli [72] attributed eating disorders, in part, to gendered societal expectations and cultural norms placed on women by patriarchal authority. Feminist scholars have since highlighted the connection between eating pathology and hierarchical aspects of the social structure [73–76]. These scholars saw women’s disturbed relationships with food and body as complex pathologies on the intersection between illness, culture and women’s psychology. In the context of self-repression, Orbach [73] pointed out that assertiveness is viewed as unfeminine and therefore undesirable in women. Lawrence & Pennycook [76] took this further, claiming that in women, “one of the central elements in anorexia is the tendency to want to please and to comply with other people’s expectations. It is when complying and pleasing others becomes incompatible with the demands of real maturity and autonomy that anorexia tends to occur” (p. 85). Regarding the demands of maturity and autonomy during adolescence, anorexia has been associated with identity crisis, and more specifically with a rejection of identity as a woman [75, 77]. Olson [78] viewed eating disorders in women and girls as compliance to socially imposed feminine ideals, which are thinner today than at any other time in history. As a final point on gender differences, sociological theories suggest that women are, in general, socialized to focus on others’ emotions more than their own and to inhibit the expression of negative emotions, so have trouble acknowledging their own experiences [79].

Taking these cultural views into account, our novel finding that the link between selflessness and eating disorder symptoms does not extend to men hardly seems surprising. Nor are our findings that for women, selflessness is significantly associated not only with disordered eating, but also with symptoms of depression, anxiety and somatization.

These results have implications for therapy and prevention. First, therapists should be aware that their patients’ tendency towards self-repression and pleasing others may be expressed in therapy, so that apparent improvements may in fact be conscious or unconscious attempts to please the therapist. Second, therapy that empowers patients characterized by self-repression, strengthens their sense of self and assertiveness, and addresses their tendency to fulfill others’ needs and desires may positively impact their symptoms of depression, anxiety, somatization and eating disorders. Yet whereas therapy that nurtures the expression of girls’ and women’s genuine needs, feelings and aspirations is likely to be helpful in their recovery from eating disorders [80], this may be less true for boys and men. For them, these therapeutic aims may be more relevant to therapy for depression, anxiety and somatization than to therapy for eating disorders. Third, therapeutic interventions for disordered eating and other internalising disorders should comprehensively address the socio-cultural context of eating problems, focusing on gender dynamics and expectations from women that weaken their voices and increase their vulnerability to social expectations and comparison with others. The focus of therapy for eating disorders and disordered eating should not be purely individualistic but address, in addition, socio-cultural factors that shape the patient’s experience. Finally, eating disorder prevention programs should address young women's loss of self and voice when they sacrifice their own interests in the context of conformity to societal beauty ideals.

The study has several limitations, which should be held in mind when considering the results. The design is cross-sectional with a single sampling point, so that the relationships observed between the study variables are associations only and no conclusions can be reached about cause and effect. A longitudinal design, with an adequate sample of both men and women, would be of great interest, and might help to unravel the differential processes for men and women. Data was collected only via self-report. Participants were community volunteers, and as always, men were more difficult to recruit than women. Findings may not extend to clinical samples of people with eating disorders.

## Conclusions

The findings of this study indicate that self-repression is more closely linked to psychopathology for women than for men. For men, self-suppression seems to be associated with symptoms of internalizing disorders, but not disordered eating. Even for women, self-repression is not connected exclusively with disordered eating, but with symptoms of psychopathology in general. Exactly why self-repression seems to be such a central and pathogenic process in women’s psychopathology but not men’s should be explored in future research. Future studies should also examine the association between self-repression and gender in more egalitarian social environments.

## Data Availability

The datasets used and analyzed during the current study are available from the corresponding author on reasonable request.

## Abbreviations

DSM-5: Diagnostic and Statistical Manual of Mental Disorders (5th ed)
MANOVA: Multivariate analysis of variance
SEM: Structural equation models
NFI: Normed fit index
RMSEA: Root mean square error of approximation
RMR: Root means square residual
